# Extraction of High Value Triterpenic Acids from *Eucalyptus globulus* Biomass Using Hydrophobic Deep Eutectic Solvents

**DOI:** 10.3390/molecules25010210

**Published:** 2020-01-04

**Authors:** Nuno H. C. S. Silva, Eduarda S. Morais, Carmen S. R. Freire, Mara G. Freire, Armando J. D. Silvestre

**Affiliations:** CICECO-Aveiro Institute of Materials, Chemistry Department, University of Aveiro, Campus Universitário de Santiago, 3810-193 Aveiro, Portugal; nhsilva@ua.pt (N.H.C.S.S.); morais.eduarda@ua.pt (E.S.M.); cfreire@ua.pt (C.S.R.F.); maragfreire@ua.pt (M.G.F.)

**Keywords:** triterpenic acids, ursolic acid, *Eucalyptus globulus* outer bark, hydrophobic deep eutectic solvents, menthol, thymol

## Abstract

Triterpenic acids (TTAs), known for their promising biological properties, can be found in different biomass sources and related by-products, such as *Eucalyptus globulus* bark, and have been extracted using organic volatile solvents such as dichloromethane. Recently, deep eutectic solvents (DES) have been identified as promising alternatives for the extraction of value-added compounds from biomass. In the present work, several hydrophobic DES were tested for the extraction of TTAs from *E. globulus* bark. Initial solubility studies revealed that DES based on menthol and thymol as the most promising solvents for these compounds given the highest solubilities obtained for ursolic acid (UA) at temperatures ranging from room temperature up to 90 °C. Accordingly, an eutectic mixture of menthol:thymol (1:2) was confirmed as the best candidate for the TTAs extraction from *E. globulus* outer bark, leading to extraction yields (weight of TTA per weight of biomass) at room temperature of 1.8 wt% for ursolic acid, 0.84 wt% for oleanolic acid and 0.30 wt% for betulinic acid. These values are significantly higher than those obtained with conventional organic solvents under similar conditions. The results obtained using these DES are promising for the recovery of TTAs for nutraceutical and pharmacological applications, while reinforcing the potential of DES as promising solvents to be applied in biorefinery processes.

## 1. Introduction

In the last decades, renewable resources have gathered a raising interest due to increasing concerns with the inevitable dwindling of fossil resources in the decades to come, as well as to the environmental impact resulting from their massive use, associated with global warming and pollution [1]. The development of value chains based on biomass, in the so-called biorefineries, requires the development of sustainable fractionation processes, allowing an integrated exploitation of all biomass fractions [2,3,4]. Furthermore, the development of biomass refining processes is also relevant to foster the valorization of side-streams and by-products of presently implemented industrial processes, while contributing to the development of the circular economy concept [5,6], fulfilling the UN Sustainable Goals [7].

The integrated exploitation of agroforest biomass and related by-products is nowadays a fundamental issue, in which the development of environmentally friendly strategies for the recovery of high-value compounds plays a significant role [2,5]. As far as forest biomass is concerned, pulp and paper industry is a key player in the development of these concepts given its importance worldwide, mainly due to the large amounts of woody biomass processed and by-products generated, and low benign technologies currently used. Furthermore, this sector needs to implement breakthrough technologies to improve efficiency, increase the products quality and portfolio, while incrasing sustainability and reducing environmental impact [8]. In particular, the pulp and paper industry generates considerable amounts of forest biomass by-products, such as leaves, branches and bark. These by-products are, in part, left in the forest for soil fertilization or are burned in pulp mills for power generation.

**Figure 1:**
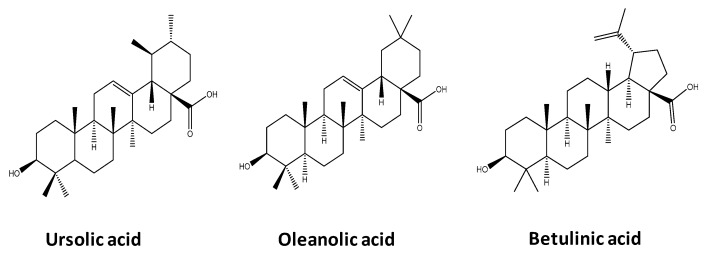
Chemical structure of the three main triterpenic acids present in *Eucalyptus* species outer bark.

*Eucalyptus* species are amongst the most commonly used species by the pulp industry in South Western Europe (Portugal and Spain), South America (Brazil and Chile), South Africa, Japan, among others [9]. *E. globulus* is the dominant species used in Portugal due to its high productivity and high quality of the pulp fibers produced [10]. However, *Eucalyptus* by-products could be further valorized if high value compounds could be extracted prior to their energy conversion. It has been reported that several *Eucalyptus* species outer barks contain large amounts of triterpenic acids (TTAs), such as ursolic (UA), betulinic (BA) and oleanolic (OA) acids [11,12,13,14] (Figure 1), which display relevant biological activities, such as antimicrobial, antitumor, hepatoprotective, anti-inflammatory, cytotoxic, anti-allergic and anti-HIV activities [15,16,17,18,19,20,21]. Accordingly, these high value compounds have been studied in a wide range of applications in the food, biomedical and pharmaceutical fields [22,23,24].

TTAs are usually extracted from biomass using volatile organic solvents, such as dichloromethane [12], *n*-hexane [25], ethanol [26] or chloroform [18], and using extraction processes as simple solid-liquid extraction and Soxhlet extraction. For instance, extraction yields of 1.4% of the three main TTAs (UA, BA, OA), from which 0.82% partakes to UA [12], have been obtained from *E. globulus* outer bark by Soxhlet extraction using dichloromethane. However, these solvents are often toxic, and some of these processes are energy demanding. Therefore, it is important to consider greener extraction technologies, where novel solvents like supercritical CO_2_ may lead to an efficient and sustainable extraction of these compounds [11,27,28]. For example, Domingues et al. [11] have optimized the extraction of TTAs from *E. globulus* outer bark using supercritical CO_2_, under 200 bar, 40 °C and 5% ethanol, but with extraction yields 20% lower than those obtained with dichloromethane [12]. Ionic liquids have also been recently studied for the extraction of TTAs, achieving higher extraction yields when compared to conventional solvents [29]. This last study opened new perspectives to study alternative and tunable solvents for the extraction of TTAs from *E. globulus* outer bark, as ionic liquids but also deep eutectic solvents (DES) [30].

A deep eutectic solvent is composed of, at least, an hydrogen bond acceptor (HBA) and an hydrogen bond donor (HBD) species, that form a eutectic mixture deviating from ideality, thus presenting a significant decrease on the melting temperature [31]. DES, if properly designed, may be considered as low-cost based solvents, and have been identified as promising environmental-friendly alternatives in extraction and conversion processes [32,33,34], preparation of innovative materials [35,36], and extraction of high-value compounds and fractionation of biomass [37,38,39,40]. A large number of stable DES based on natural compounds, such as organic acids, amino acids and sugars, called natural deep eutectic solvents (NADES), have also been recently described [41].

**Figure 2:**
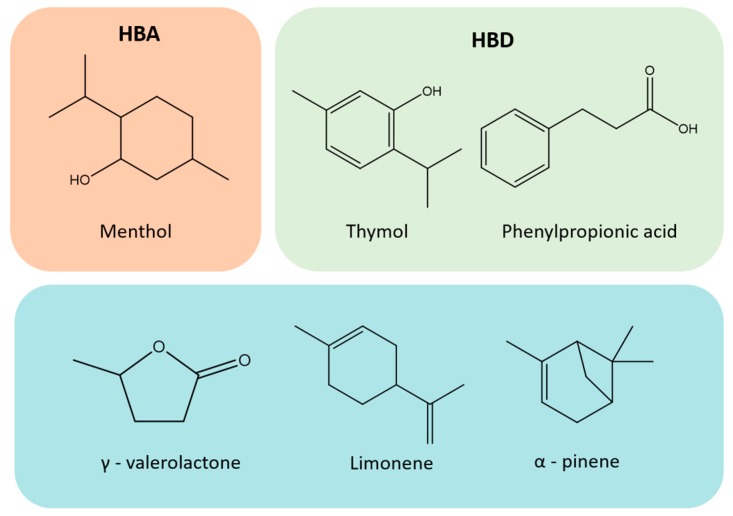
Chemical structures the individual components of the deep eutectic solvents (DES) prepared, as well as other bio-based solvents used in this work.

Based on the exposed, and given our interest on the exploitation of triterpenic acids from *E. globulus* outer bark [11,12,28,42], herein we investigated hydrophobic (NA)DES for the extraction of TTAs. TTAs have a negligible solubility in water (1.02 × 10^−7^ g/L) [43], and as mentioned above, their extraction is usually performed with organic solvents. Nevertheless, the solubility of UA in some of these solvents is not higher than 15 mg per g of solvent [44,45]. Thus, given the hydrophobic nature of TTAs, hydrophobic NADES can be envisioned as promising alternative solvents. In this work, menthol is considered the HBA while thymol and phenylpropionic acid were the HBDs (Figure 2). Firstly, this study comprised a solubility study of ursolic acid (the most abundant TTA in *E. globulus* outer bark) in the selected NADES, at different molar ratios and temperatures, followed by the application of the optimal conditions to the extraction of TTAs from *E. globulus* outer bark. To demonstrate the improved efficiency provided by NADES in the UA solubility studies, the individual components of the studied NADES were also investigated, along with γ-valerolactone, limonene and α-pinene (Figure 2), which are bio-based solvents that have already demonstrated their efficiency in the extraction of triterpenes from other biomass sources [46]. The extractions were optimized regarding the DES molar ratio, biomass/DES ratio and temperature.

Briefly, pure UA was added in excess amounts to the different solvents chosen and samples were kept under constant agitation at different temperatures (room temperature—RT, 60, 75, 90 °C), in order to identify the best candidate for the TTAs extraction from *E. globulus* outer bark. Afterwards, solid–liquid extraction of TTAs from *E. globulus* outer bark was carried out at 500 rpm during 4h at given temperatures. The experiments were prepared by adding 200 ± 3 mg of *E. globulus* outer bark to 3.0 ± 0.1 g of solvent. Given the results of the solubility tests, extraction studies were performed using the eutectic mixture menthol:thymol (1:2). Extractions using different biomass/DES ratios from 0.025 to 0.15 were further conducted at RT, 60 and 90 °C. Three major TTAs have been identified, namely ursolic acid (UA), oleanolic acid (OA), and betulinic acid (BA), which were quantified by high performance liquid chromatography with diode array detection (HPLC-DAD).

## 2. Results and Discussion

### 2.1. Solubility of Ursolic Acid

With the goal of selecting a sustainable solvent with high capability for solubilizing, and therefore extracting, triterpenic acids (TTAs), several bio-based solvents and NADES were screened in solubility tests using ursolic acid (UA) as a model TTA since it is the most abundant TTA in *E. globulus* outer bark. The solubility of UA was measured in several bio-based molecular solvents, namely limonene, menthol, thymol, γ-valerolactone, and α-pinene, as well as in menthol-based natural deep eutectic solvents (NADES) with phenyl propionic acid (PPA) and thymol in a molar ratio of 1:1. Considering the melting points of menthol and thymol, 36 °C and 49 °C, the preliminary solubility tests were carried out at 60 °C in order to obtain comparable results between all solvent systems (including the individual components of NADES) and NADES. However, it should be remarked that all NADES studied are liquid at room temperature.

**Figure 3:**
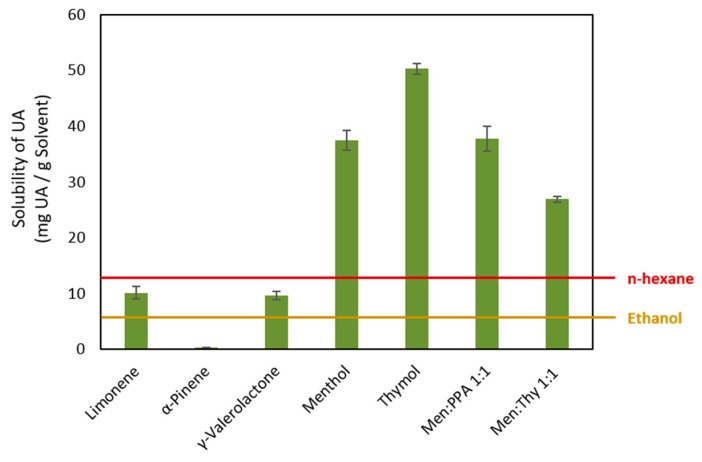
Solubility of ursolic acid (UA) in different solvents: limonene, α-pinene, γ-valerolactone, menthol, thymol, menthol:phenyl propionic acid (PPA) (Men:PPA), menthol:thymol (Men:Thy) at 60 °C; comparison with some volatile organic solvents: n-hexane and ethanol.

According to the obtained results (Figure 3), UA shows higher solubilities in most of the solvents tested than the values reported for common organic solvents. For instance, the solubility of UA in n-hexane is ca. 13 mg/g solvent and in ethanol is ca. 5 mg/g [45]. Using limonene and γ-valerolactone, similar solubilities for UA were obtained (~10 mg/g). Significantly higher solubilities of UA were obtained in menthol (37 ± 2 mg/g), thymol (50 ± 1 mg/g) and menthol-based DES, namely menthol:PPA acid 1:1 (38 ± 2 mg/g) and menthol:thymol 1:1 (28 ± 3 mg/g). Overall, thymol is the solvent allowing the highest solubility of UA, allowing a 4- and 10-fold increase in solubility when compared to n-hexane and ethanol, respectively.

**Figure 4:**
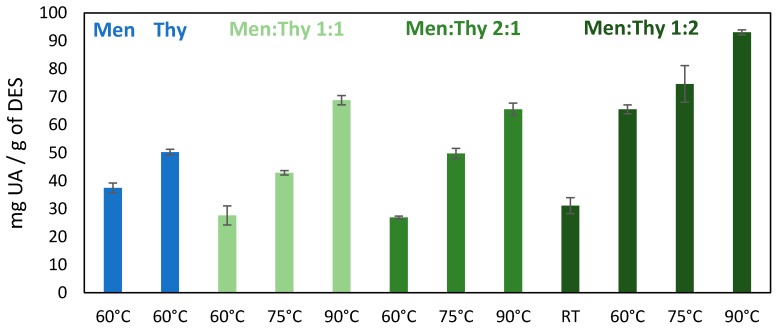
Solubility of ursolic acid (UA) in menthol and thymol at 60 °C and in menthol:thymol natural deep eutectic solvents (NADES) in different proportions (1:1, 1:2, 2:1) at different temperatures (RT, 60, 75, 90 °C).

Since pure thymol has shown the highest solubility of UA, followed by menthol and the menthol:thymol 1:1 NADES, a study on the effect of different ratios of the two monoterpenes in the NADES composition was conducted, according to solid-liquid phase diagrams reported elsewhere [47,48]. Specifically, the solubility of UA was carried out in NADES with two different molar ratios of menthol:thymol (namely, 1:2 and 2:1), at different temperatures (RT, 60, 75 and 90 °C), whose results are shown in Figure 4.

The results obtained show that the solubility of UA is higher in the NADES with the higher thymol ratio (menthol:thymol 1:2). In fact, a remarkable increase in the solubility of UA was observed by conjugating menthol and thymol at 60 °C, in a 1:2 ratio, leading to a UA solubility of 66 ± 2 mg/g when compared to its solubility in the individual components, namely menthol (37 ± 2 mg/g) and thymol (50 ± 1 mg/g), and in the NADES menthol:thymol at the molar ratios 1:1 and 2:1, with 28 ± 3 mg/g and 27 ± 0.5 mg/g, respectively.

By increasing the temperature, an increase in the solubility of UA was obtained, with values in solubility higher than 70 mg/g. A maximum solubility of UA of 93 ± 1 mg/g was achieved at 90 °C for the menthol:thymol 1:2 NADES.

Overall, the menthol:thymol 1:2 NADES performs better than the corresponding individual components in the solubilization of UA, and the highest solubility of UA was observed at 90 °C. Furthermore, the UA solubility in this NADES is remarkably higher than in conventional organic solvents as dichloromethane or ethanol (Figure 3). Therefore, these results open a new and promising solution for the extraction of triterpenenic acids from different biomass sources and from *E. globulus* outer bark in particular.

### 2.2. Extraction of TTAs from E. globulus Bark

After studying the solubility of UA in several solvents and after identifying the most promising solvent and temperature, extraction assays were performed with the NADES menthol:thymol (1:2) at 90 °C. The three main TTAs present in the *E. globulus* outer bark biomass, namely ursolic acid (UA), oleanolic acid (OA) and betulinic acid (BA), were identified and quantified by HPLC-DAD using pure standards as reference for retention time and quantification.

**Figure 5:**
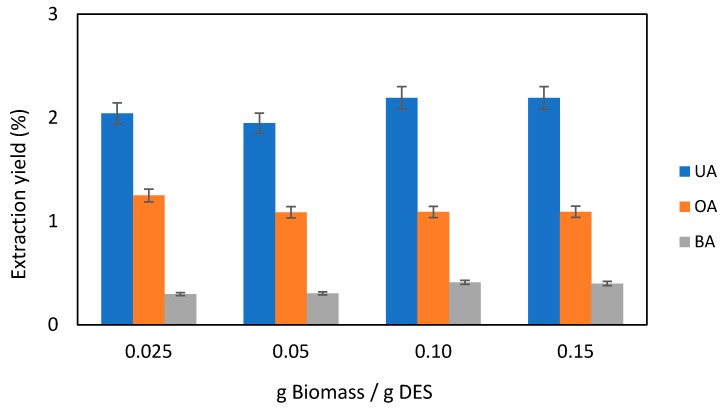
Extraction yields of ursolic (UA), oleanolic (OA) and betulinic (BA) acids, using different ratios of biomass/solvent at 90 °C.

First, the biomass/DES ratio was studied in order to appraise the maximum quantity of biomass that could be used without compromising the extraction yields due to mass transfer or saturation effects, going from 0.025 up to 0.15 g of dried biomass/g of solvent, at the following fixed conditions: 90 °C; stirring of 500 rpm, 4h. It was found that above 0.15 g/g the extraction medium becomes highly viscous and difficult to operate. From the results depicted in Figure 5, the total TTAs extraction yield ranges between 3.4 and 3.7 wt%, which is significantly higher than those obtained previously with supercritical CO_2_ (1.2%) [11] and dichloromethane (1.4%) [12] from the same biomass. Furthermore, the extraction yields are not compromised by the increased amount of biomass up to 0.15 g/g, meaning higher amounts of TTAs extracted per mass of solvent, a crucial factor for implementing efficient industrial processes. In terms of each TTA, UA was, as expected, the most abundant component (2.0–2.2 wt%), followed by OA (1.1–1.3 wt%) and BA (0.30–0.41 wt%). An illustrative HPLC chromatogram corresponding to TTAs rich extract obtained using the NADES menthol:thymol 1:2 at 90 °C (0.15 g/g) is provided in Figure S1 (Supplementary Materials).

**Figure 6:**
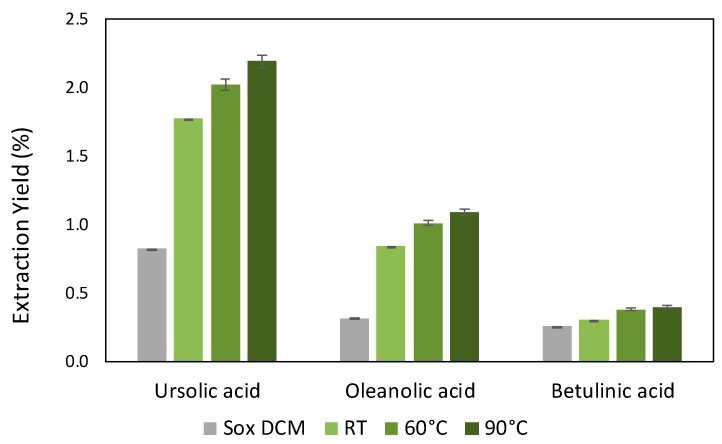
Extraction yields of ursolic, oleanolic and betulinic acids, using Soxhlet extraction with dichloromethane, and simple solid–liquid extraction with the NADES menthol:thymol 1:2 (0.15 g biomass/g DES), at room temperature (RT), 60 and 90 °C.

Based on the previous set of results, the 0.15 ratio was then chosen to carry out extraction assays varying the temperatures (RT, 60 and 90 °C), whose results are shown in Figure 6. Comparing these results with those obtained by Soxhlet extraction with dichloromethane, there is an increase of 1.4, 2.2 and 0.5-fold in the extraction of UA, OA and BA, respectively, performing the extraction at 90 °C. By decreasing the temperature down to 60 °C, extraction yields of 2.0 ± 0.1 wt% (UA), 1.0 ± 0.1 wt% (OA) and 0.38 ± 0.02 wt% (BA) were obtained, with a total TTAs extraction yield of 3.4 wt%. Additionally, performing the extraction at RT, lower extraction yields are obtained, namely of 1.8 ± 0.1% for UA, 0.84 ± 0.04% for OA and 0.30 ± 0.01% for BA, with a decrease to 2.9% of the total TTAs extracted. In summary, higher temperatures are slightly more favorable for the extraction of TTAs from the studied biomass, which may be related to the fact that higher temperatures lead to higher solubilities, as shown in Figure 4, and lead to a lower solvent viscosity and to lower mass transfer constraints. However, the extraction yields seem to be mainly limited by the maximum amount of TTAs in the biomass since the amounts extracted are well below the solubility of TTAs in the solvent (the maximum of 2.2 wt% of extraction yield (90 °C, 0.15 ratio) corresponds to 4.6 mg of TTAs/g of NADES).

Although higher extraction yields were obtained at higher temperatures, the total TTAs extraction yield obtained at RT using the NADES menthol:thymol (1:2), without any source of heating, is still one-fold higher comparing to the one obtained using a Soxhlet extraction with dichloromethane (Figure 6), showcasing the outstanding capability of appropriate hydrophobic DES to extract TTAs. These results suggest that performing the extraction of TTAs at RT may be a more relevant strategy to be applied at a large-scale level since it corresponds to a lower energetic input. Moreover, given the natural origin of the solvent used, these results lead us to envision on the one hand the possibility of directly using these extracts without removing the solvent in specific (e.g., topical) applications. Finally, further studies will be necessary on the recovery of the TTAs from the NADES extracts and to fractionate them into pure compounds for more demanding applications.

## 3. Materials and Methods

### 3.1. Materials

Ursolic (UA), oleanolic (OA) and betulinic (BA) acid standards, with a purity higher than 98 wt%, were acquired from Chemos GmbH (Germany). Menthol and thymol were acquired from Acros Organics (USA), and γ-valerolactone, α-pinene, and phenyl propionic acid were acquired from Sigma (USA). The mobile phase used in the HPLC analysis was composed of methanol (purity ≥ 99.99 wt%) from VWR Chemicals (USA), trifluoroacetic acid (TFA) from Sigma (USA) and ultrapure water (purity ≥ 99.99 wt%) from Merck (Germany), both HPLC grade. *Eucalyptus globulus* outer bark was manually collected from an experimental plantation and dried at room temperature. Representative samples of each bark fraction were collected, ground and sieved, and the granulometric fractions of 40–60 mesh (representing more than 90% of the sample) were used in the assays.

### 3.2. DES Preparation

The humidity of the different DES precursors (HBA and HBD) was taken in account regarding their preparation and was measured using a Metrohm 831 Karl Fisher coulometer. The different NADES were prepared by mixing the precursors at the desired molar ratios in sealed glass vials with constant stirring and heated, until a homogeneous and transparent liquid was formed (maximum temperature of 80 °C), kept for one hour at this temperature and then cooled down to room temperature. Given the hydrophobic nature of TTAs, hydrophobic NADESs based on menthol, thymol and phenylpropionic acid were investigated and prepared in different molar ratios: menthol:propionic acid 1:1 and menthol:thymol 1:1, 1:2 and 2:1.

### 3.3. Ursolic Acid Solubility Tests

Since ursolic acid (UA) is the main triterpenic acid present in *E. globulus* [12], it was selected out of the three TTAs to perform solubility studies in the different solvents, namely limonene, menthol, thymol, γ-valerolactone, and α-pinene, and menthol-based NADES with PPA and thymol, in a 1:1 ratio. Pure UA was added in excess amounts to the different solvents chosen and samples were kept under constant agitation at different temperatures (RT, 60, 75, 90 °C). Previously optimized equilibration conditions were established as follows: stirring velocity of 750 rpm and equilibration time of, at least, 72 h. At least three independent samples were prepared for the determination of the average solubility value and respective standard deviation. After saturation of the solutions, and complete sedimentation of the UA excess using a Megafuge 16 R centrifuge (Thermo Scientific, Waltham, MA, USA), a 200 μL aliquot was taken, diluted with 800 μL of methanol, and carefully filtered with a 0.20 μm syringe filter. The quantification of TTAs in each solution was carried out by high performance liquid chromatography with diode array detection (HPLC-DAD) (Shimadzu, model Prominence), using an analytical C18 reversed-phase column (250 × 4.60 mm), Kinetex 5 μm C18 100 Å, from Phenomenex (Torrance, CA, USA). The mobile phase consisted of 87% (*v*/*v*) methanol, and 13% (*v*/*v*) of an aqueous solution of 0.1% (*v*/*v*) TFA. Separations were conducted in isocratic mode, at a flow rate of 1 mL min^−1^ and with an injection volume of 10 μL [29]. The wavelength was set at 210 nm. Each sample was analyzed, at least, three times. The column oven and the autosampler were kept at 30 °C.

### 3.4. Extraction of TTAs from Eucalyptus globulus Bark

Solid–liquid extraction of TTAs from *E. globulus* outer bark was carried out using a commercial carousel from Radleys Tech that is able to both stir and maintain the temperature within ± 0.5 °C. In all experiments the stirring was kept at 500 rpm, and the extraction was conducted during 4h. The experiments were prepared by adding 200 ± 3 mg of *E. globulus* outer bark to 3.00 ± 0.05 g of solvent. Given the results of the solubility tests, extraction studies were performed using the eutectic mixture menthol:thymol (1:2), using the best conditions obtained in such tests with UA, at 90 °C, and varying the biomass/DES ratio from 0.025 to 0.15. For comparative purposes, extraction assays were also carried out at RT and 60° C. The yields of Soxhlet extraction with dichloromethane, for the 3 TTAs were also determined for comparison purposes.

After the extraction step, extract solutions were separated from the biomass by centrifugation (at 4000 rpm for 30 min using an Eppendorf centrifuge 5804), the supernatant was filtered using a 0.20 μm syringe filter. A 50 mg aliquot was taken, mixed with 950 μL of methanol, and filtered through a 0.2 μm syringe filter, and TTAs content were quantified by HPLC-DAD as described above for solubility assays. Three major TTAs have been identified in the extracts according to the respective standards and retention-time values: ursolic acid (UA), oleanolic acid (OA) and betulinic acid (BA). A calibration curve was previously established for each TTA (Table 1). TTAs extraction yields are expressed as the percentage ratio between the weight of each TTA and the total weight of the dried biomass.

## 4. Conclusions

Facing the need of sustainable solvents and processes, herein we investigated the potential of some bio-based solvents and NADES as alternative solvents over the commonly used volatile organic solvents for the extraction of TTAs from *E. globulus* outer bark. Among the solvents tested, menthol and thymol lead to solubilities of ursolic acid higher than those achieved with conventional volatile organic solvents (>15 mg UA/g solvent, at 60 °C). Moreover, at 90 °C, the eutectic mixture menthol:thymol in a molar ratio of 1:2 promotes a significant increase in the solubility of ursolic acid, 93 ± 1 mg/g. After identifying the best solvent, extraction assays from *E. globulus* outer bark biomass were conducted with the same NADES at RT, 60 °C and 90 °C, leading to promising yield extractions for the three TTAs studied: ursolic acid (*ca.* 2 wt%), oleanolic acid (*ca.* 1 wt%) and betulinic acid (*ca.* 0.4 wt%). By decreasing the temperature down to room temperature, 2.9 wt% total TTAs extraction yield was obtained, a result not significantly lower than at 90 °C, while contributing to a lower energetic input. Therefore, from a large-scale point of view, the extraction of TTAs at room temperature is the best strategy. It is expected that the results obtained contribute to span the application of NADES to extract high value compounds from biomass by-products, while reinforcing their potential in biorefinery and circular economy concepts development.

## Figures and Tables

**Table 1:** Calibration curves of the three triterpenic acids studied, and the respective retention times (min) and the coefficients of correlation (R^2^).

	Retention Time (min)	Calibration Curve	R^2^
UA	11.2	*y* = 4487847.76*x* + 2541.49	0.997
BA	10.2	*y* = 4175298.99*x* − 7379.03	0.995
OA	10.7	*y* = 3664880.72*x* − 98403.86	0.991

## Supplementary Materials

Figure S1. Illustrative HPLC chromatogram corresponding to the extraction of TTAs using the NADES menthol:thymol 1:2 at 90 °C (0.15 S/L ratio).
