# Supplementary material for: Extraction of High Value Triterpenic Acids from Eucalyptus globulus Biomass Using Hydrophobic Deep Eutectic Solvents
Source: Molecules. 2020 Jan 4;25(1):210. doi: 10.3390/molecules25010210 (PMC6983165; doi:10.3390/molecules25010210)
Supplement: Supplementary file 1 [file molecules-25-00210-s001.pdf]

Supplementary Materials

Article

Extraction of high value triterpenic acids from *Eucalyptus globulus* biomass using hydrophobic deep eutectic solvents

Nuno H. C. S. Silva, Eduarda S. Morais, Carmen S. R. Freire, Mara G. Freire and Armando J. D. Silvestre*

CICECO-Aveiro Institute of Materials, Chemistry Department, University of Aveiro, Campus Universitário de Santiago, 3810-193 Aveiro, Portugal
* Correspondence: armsil@ua.pt

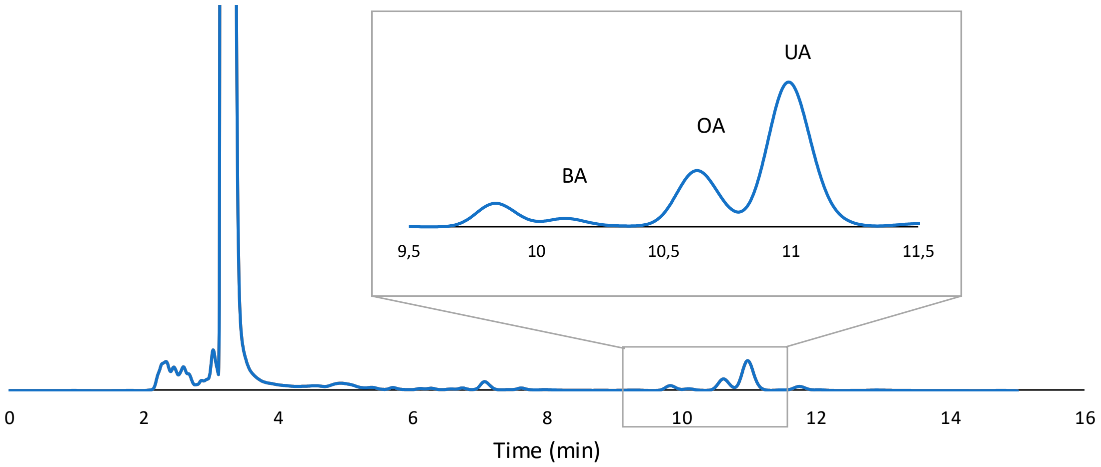

Figure S1. Illustrative HPLC chromatogram corresponding to the extraction using the NADES menthol:thymol at a molar ratio of 1:2 at 90°C (0.15 S/L ratio).
